# Numerical Design of Microporous Carbon Binder Domains Phase in Composite Cathodes for Lithium-Ion Batteries

**DOI:** 10.1021/acsami.3c00998

**Published:** 2023-05-31

**Authors:** Ruihuan Ge, Adam M. Boyce, Yige Sun, Paul R. Shearing, Patrick S. Grant, Denis J. Cumming, Rachel M. Smith

**Affiliations:** † Department of Chemical and Biological Engineering, The University of Sheffield, Sheffield S10 2TN, UK; ‡ Electrochemical Innovation Lab, Department of Chemical Engineering, University College London, London WC1E 7JE, UK; § Department of Materials, University of Oxford, Oxford OX1 3PH, UK; ∥ School of Mechanical and Materials Engineering, University College Dublin, Dublin 4, Ireland; ⊥ The Faraday Institution, Quad One, Harwell Science and Innovation Campus, Didcot OX11 0RA, UK

**Keywords:** lithium-ion battery (LIB), carbon binder domain (CBD), electrode microstructure, stochastic methods, microporosity

## Abstract

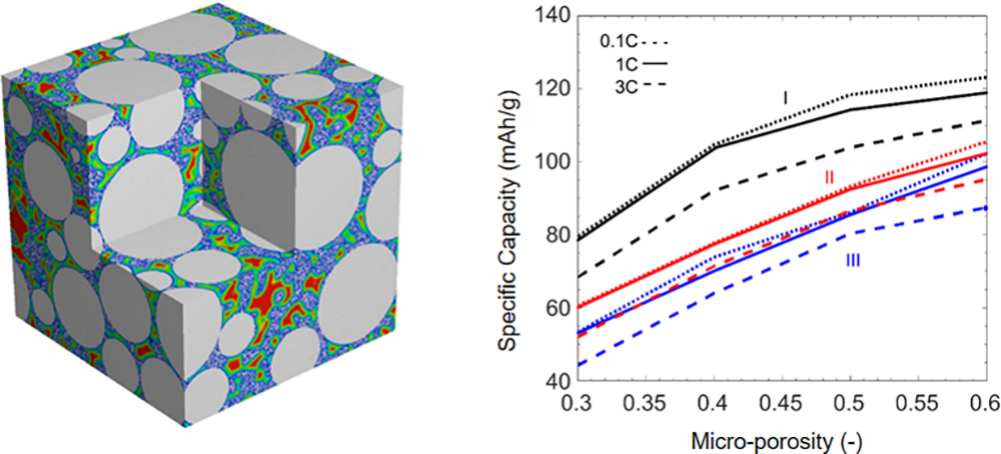

Lithium-ion battery (LIB) performance can be significantly affected by the nature of the complex electrode microstructure. The carbon binder domain (CBD) present in almost all LIB electrodes is used to enhance mechanical stability and facilitate electronic conduction, and understanding the CBD phase microstructure and how it affects the complex coupled transport processes is crucial to LIB performance optimization. In this work, the influence of microporosity in the CBD phase has been studied in detail for the first time, enabling insight into the relationships between the CBD microstructure and the battery performance. To investigate the effect of the CBD pore size distributions, a random field method is used to generate in silico a multiple-phase electrode structure, including bimodal pore size distributions seen in practice and microporous CBD with a tunable pore size and variable transport properties. The distribution of macropores and the microporous CBD phase substantially affected simulated battery performance, where battery specific capacity improved as the microporosity of the CBD phase increased.

## 1 Introduction

Lithium-ion batteries (LIBs) are widely used in a range of sectors including electric vehicles and grid-scale storage because of their combination of decreasing cost, high specific energy density, and reasonable lifetime. Nonetheless, further cost reductions and lifetime increases, along with improvements in power density, are required.^1^ The LIB anode and cathode are multi-material, porous composites and their microstructure can significantly affect battery performance.^2−5^ For example, the LIB cathode microstructure consists of (i) the majority active material (AM) particle phase for lithium-ion storage, (ii) the carbon binder domain (CBD) that is used to facilitate electronic conduction and ensure mechanical rigidity, and (iii) interconnected, tortuous porosity (30–40 vol %) that is filled with the Li-ion-containing electrolyte. The CBD is typically located at AM particle contacts and is itself a composite that comprises conductive additives (e.g., carbon black) and a nonconductive polymeric binder such as polyvinylidene fluoride (PVDF).^6^

The final microstructure and performance of a LIB electrode is a function of the optimization of the fractions of each of three phases (i)–(iii) above, plus the integrated effects of a multiplicity of process parameters in each of the key stages of electrode fabrication: mixing and slurry formation, deposition, drying, and calendering.^7^ Each individual manufacturing step can substantively affect the AM particle arrangement and CBD distribution within an electrode.

Once the electrode is processed further into the LIB and charge/discharge takes place, complex Li-ion and electron transport processes occur within the electrode microstructure. While Li-ion transport occurs in the electrolyte that fills the interconnected pores and into and within the AM particulates, the CBD provides the electronic conduction pathways and plays a key role in the overall electrochemical properties and capacity fading.^2,8^ Therefore, there is an ongoing interest in quantifying the contribution of the different phases, as a function of morphology and fraction, to the LIB performance in both well-characterized experimental and artificially generated electrode microstructures.^9^

The exploration of CBD effects has been relatively neglected in comparison with the effects of porosity and the AM and in particular relates to difficulty in quantifying CBD characteristics in electrodes where the CBD fraction may be less than 10 vol %. While X-ray computed tomography (XCT) has proved to be a useful tool to distinguish the AM particle skeleton, for example, in LiNi_*x*_Mn_*y*_Co_*z*_O_2_ (NMC)-based electrodes,^10^ non-active phases and particularly the micro- and nanoscale CBD phase are nontrivial to distinguish via XCT. Alternatively, using focused ion beam-scanning electron microscopy (FIB-SEM), Zielke et al.^11^ characterized the porous CBD phase but this process is time-consuming and destructive of the electrode.

The advent of nano-CT has allowed pore size distributions and volume fractions of the CBD phase to be measured quantitatively^6^ and the reconstructed CBD phase related to battery performance.^12,13^ The convoluted CBD phase network influences the Li^+^ and electron transport through tortuous porosity pathways. A dual-scan superposition approach by XCT/nano-CT has been proposed by Lu et al.^13^ to reconstruct three-dimensional (3D) electrode structures that can resolve the nanoscale CBD phase over the length scale of the electrode thickness. The effect of microstructural heterogeneities on the battery performance was then evaluated by electrochemical analysis.^14^

In addition to X-ray tomography, algorithms have been developed to artificially reconstruct or generate CBD phase and granular microstructures. The microstructure–property relations can be assessed by various types of numerical simulations. The discrete element method (DEM) has been used to model granular microstructures and corresponding transport properties.^3,15,16^ By using a resistor network method, Birkholz et al. investigated the effective conductivity of granular electrode structures considering the pore phase and overlapping spheres, leading to a better understanding of granular microstructures on effective transport properties.^17,18^ Using high-fidelity DEM simulations, Srivastava et al. showed that electrode microstructures with tailored transport properties can be generated by controlling the CBD cohesive forces and AM-CBD adhesive forces.^19^ Zielke et al.^20^ reconstructed 3D battery cathodes by combining the AM phase characterized by XCT and used two models—a random cluster model and a fiber model—to generate a virtual CBD phase. They confirmed a strong influence of the CBD morphology and volume fraction on electrode ionic and electronic parameters. Mistry et al.^21^ developed an interfacial energy-based approach to control the effect of long-range and short-range transport properties within battery electrodes. Recently, a level-set approach has been proposed by Trembacki et al.^3,22^ to generate a bridge-like synthetic CBD phase between particles. They then further investigated the transport properties of these electrode structures under different calendering conditions.^23^ A recent work proposed by Usseglio-Viretta et al.^24^ compared the effective transport coefficients of uniform and heterogeneous CBD phase distributions, and the role of carbon-binder weight loading on the battery performance was investigated. Overall, virtual CBD algorithms can provide insight into the ways CBD affects electrode performance, enabling investigation of morphology, bridging, coating, distribution, etc.^19,25,26^

However, the character and effects of an artificially generated CBD are difficult to compare and validate via experimental characterization as most CBD algorithms are based on qualitative morphological descriptions. In addition, the CBD contains submicron scale features that cannot be captured by these algorithms.^2^ To address these issues, a new CBD phase algorithm is proposed. The effects of changes in the CBD network on the battery performance are then explored by numerical modeling. The novelty of the algorithm and the corresponding numerical modeling work is (i) a description of microporous CBD generated using a thresholding random field approach; (ii) synthetic electrode structures with bimodal pore size distributions validated by experimental characterization; and (iii) electrode structures that consider different CBD phase distributions and calendering conditions, and the strong resulting effect on electrode transport properties evaluated by electrochemical modeling. To the best of the authors’ knowledge, this is the first time that the character of the microporous CBD phase has been captured appropriately into battery performance simulations, using a methodology that allows for flexible and realistic manipulations of CBD properties across a wide range.

## 2 Methodology

### 2.1 Experimental Methods

The electrodes used for SEM analysis were prepared with the LiNi_0.6_Co_0.2_Mn_0.2_O_2_ (NMC622, BASF), C65 carbon black (Imerys), and PVDF binder (Solvay) in the weight ratio of 96:2:2. A dual-beam Xe^+^ ion plasma focused ion beam (Thermo Scientific Helios G4 PFIB CXe DualBeam) was used for electrode cross-sectioning and imaging. A platinum protection layer was deposited on the top of the electrode surface to reduce the curtaining effect. Secondary electron images of both as-cast and calendered electrodes were taken at 10 kV (1.6 nA) by an Everhart–Thornley detector.

### 2.2 Computational Methods

#### 2.2.1 Structure Generation and Analysis

The AM particle positions of calendered electrode structures with different volume fractions φ_Par_ were generated using particle packing algorithms in Altair EDEM. MATLAB programming was used to generate the microporous CBD phase. The pore size distribution of different generated structures was calculated using the PoroDict module in GeoDict software. The data was further analyzed via MATLAB programming.

#### 2.2.2 Electrical Conductivity and Tortuosity

The electrical conductivity and tortuosity were determined by solving the Poisson equation. These calculations were performed using GeoDict and TauFactor. In this work, the electrical conductivity of discharged AM particles is σ_0, AM_ = 0.00016 S/m, and the intrinsic electrical conductivity of the CBD phase is σ_0, CBD_ = 1490 S/m.^11,27^ By inputting the CBD phase volume fraction φ and tortuosity τ, the effective CBD phase electrical conductivity σ_eff_ can be estimated using τσ_eff_φ^–1^ = σ_0_. The calculated effective conductivity of the CBD phase with the volume fraction φ = 0.5 is about 375 S/m, which is in the range reported in literatures.^11,28^

#### 2.2.3 Electrochemical Modeling

Simpleware ScanIP was used to mesh the generated microstructures, giving approximately 3.5 million linear tetrahedral elements, with 1.8 million degrees of freedom. The theoretical framework, which is outlined in the Supporting Information (Figure S2 and Tables S7–S9), was implemented in the finite element software COMSOL Multiphysics (v5.6, Sweden) using a 3D tomography-based mesh as described. The Parallel Direct Sparse Solver (PARDISO) was used to solve the discretized transport and electrode kinetics equations. A segregated approach was taken, which involved solving the coupled field variables in a sequential, staggered way. Time stepping was handled using second-order backward Euler differentiation. Further details on the methodology can be found in ref (29).

## 3 Experimental Characterization and Model Description

### 3.1 Experimental Characterization and Structure Generation Overview

**Figure 1:**
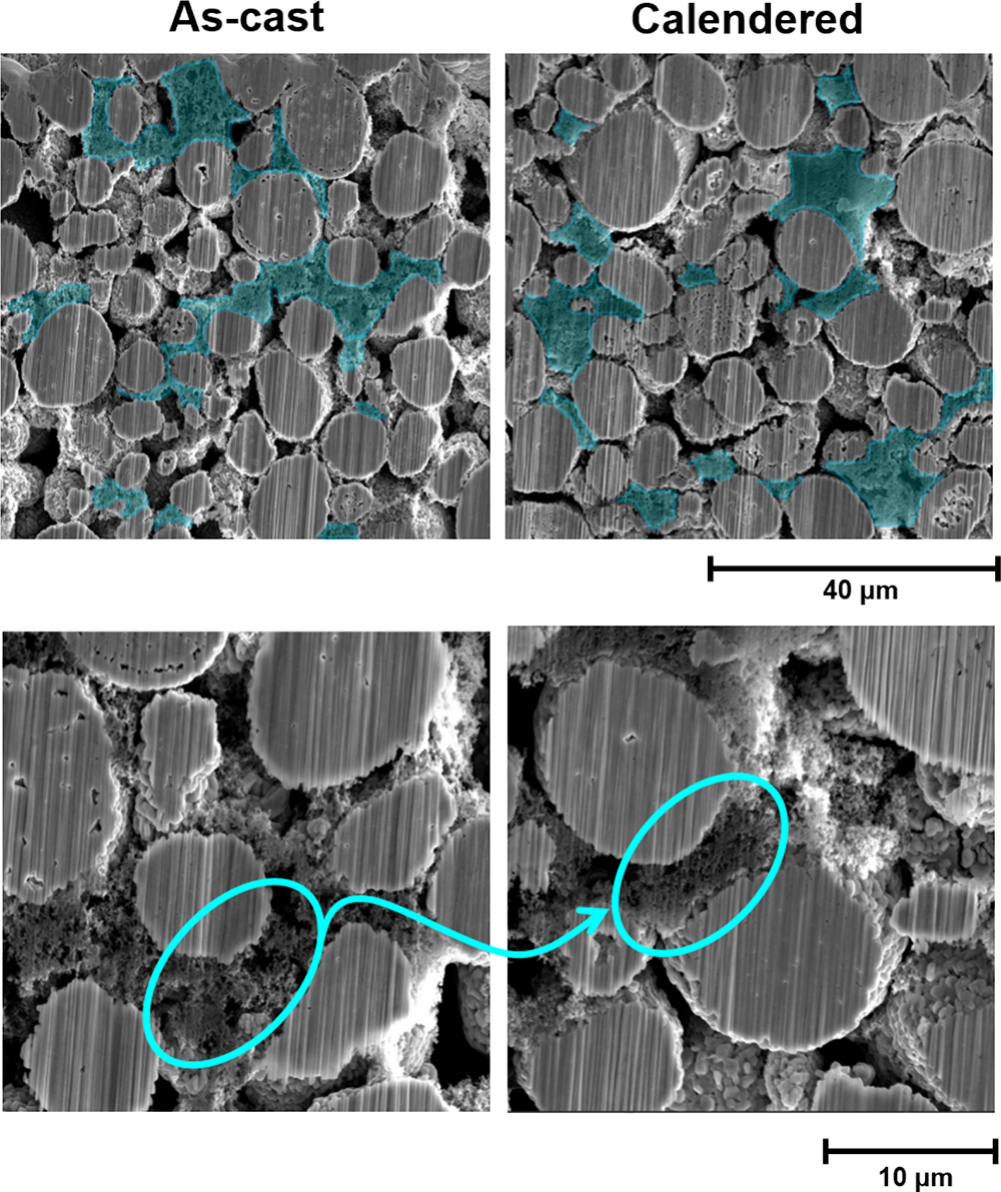
SEM images of as-cast and calendered NMC622 cathode structures (top, CBD phase distribution; the CBD phase is colored in blue; bottom, magnification of the porous CBD phase).

Figure 1 presents the high-resolution SEM images of as-cast and calendered LiNi_0.6_Mn_0.2_Co_0.2_O_2_ (NMC622) cathode cross-sectional structures, where large micrometer-scale macropores and submicron microporous CBD phases can be distinguished. The microporous CBD phase (highlighted in blue) is distributed in the void space between AM particles and forms a conductive network across the electrode. Subsequent calendering is used to densify the structure and results in a reduction of the macropore size. Higher-magnification SEM images in Figure 1 show the porosity within the CBD at a submicron scale. In general, the CBD phase itself is also somewhat densified by calendering, with a reduction in the number of micron-scale pores. Nonetheless, even after calendering, the electrode contains both micron-sized macropores between AM particles and submicron micropores within the CBD phase.

**Figure 2:**
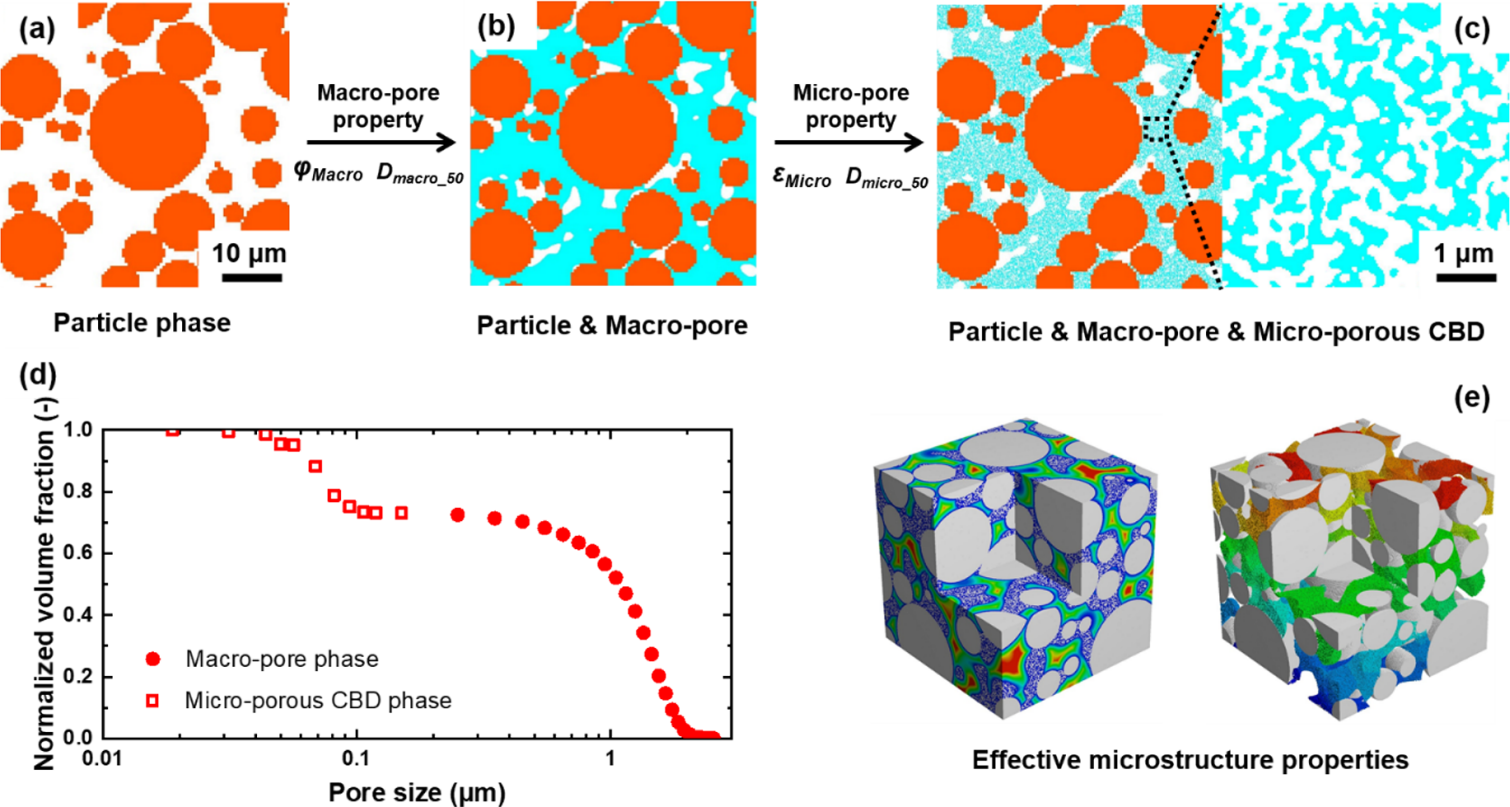
Overview of the structure generation with a bimodal pore size distribution: (a) particle phase, (b) particle and macropore phase, (c) structure considering the submicron microporous CBD phase, (d) resulting bimodal pore size distribution of the 3D synthetic electrode structure, and (e) effective microstructure properties.

To capture the microstructural complexity of these structures, Figure 2 shows schematically the approach designed to generate electrode structures that describe the AM particle phase, macropore phase, and microporous CBD phase (Figure 2a–c, respectively). Macropores and microporous CBD phases were generated separately by using a thresholding random field algorithm. Using the calculated macropore phase volume fraction φ_Macro_ and macropore size *D*_Macro_50_, the macropores with the homogenized CBD phase were generated between AM particles (Figure 2b). 3D microporous CBD structures were further generated by using CBD phase microporosity ε_Micro_ and micropore size *D*_Micro_50_ (Figure 2c). The 3D synthetic electrode structure has a tunable bimodal pore size distribution, as shown in Figure 2d, which is comparable with the experimental characterization results. Afterward, effective microstructure properties such as tortuosity and electrical conductivity were calculated (Figure 2e). By using this approach, physical properties at a micropore scale within the CBD phase could be visualized. Alternatively, by evaluating the effective properties of generated microporous CBDs, we can also use the electrode structure with macropores and homogenized properties (Figure 2b) in simulations to reduce computational resources. The detailed algorithms used for the microstructure generation are described in the following subsections.

### 3.2 Microstructure Algorithm Description for CBD Phase Generation

A thresholding random field approach was considered to facilitate the generation of the microstructures. Gaussian random fields *T*(***x***) were constructed according to refs (30, 31). The mean of the Gaussian random field is zero, and the corresponding covariance function *C*(***x***, ***y***) can be defined as
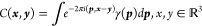
1where ( · , · ) denotes the Euclidean inner product and γ(***p***) is the spectral density of this Gaussian random field. γ(***p***) is defined as
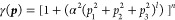
2where α, *l*, and *n* are used to control the length scales of the generated microstructures.

For constructing the Gaussian random field of *N*^3^ voxels, a 3D array *W* in which all elements are independent and normally distributed is first generated. Using forward Fourier transform  and inverse Fourier transform , the Gaussian random field *T*(***x***) can be obtained as follows:
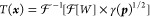
3

A porous material with a prescribed porosity ε can be generated using a threshold β. The generated microstructure Φ(***x***) can be described using the following function:
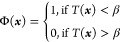
4

**Figure 3:**
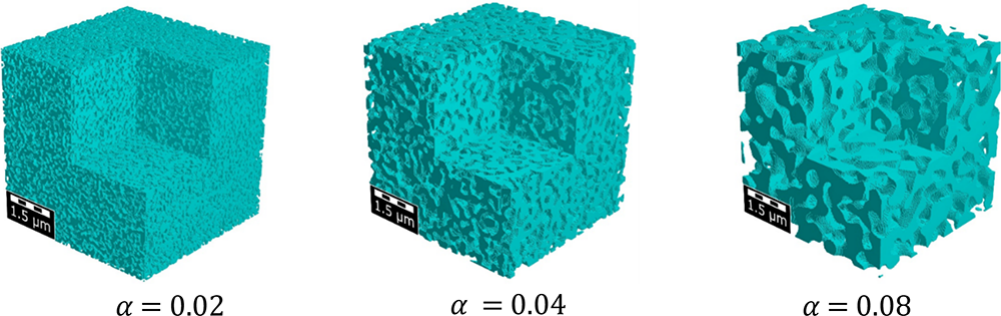
Generated porous microstructures considering different length scales α when porosity ε = 0.5.

**Figure 4:**
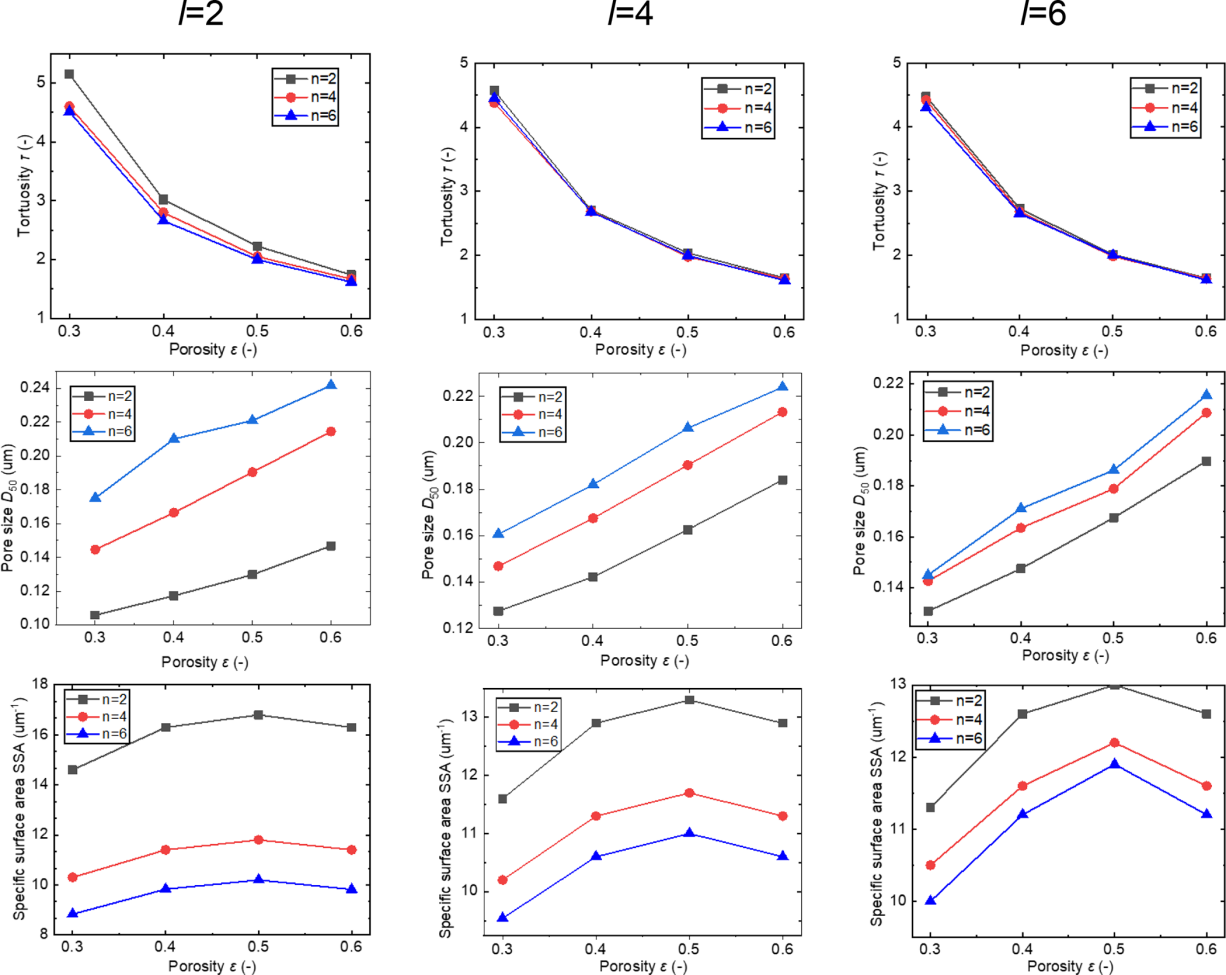
Tortuosity, average pore size, and SSA of different stochastic microstructures and the porous CBD phase with different length scales *l* and *n* when the length scale α = 0.04.

Using this approach, microstructures with desired properties can be generated by incorporating different length scales. For a microstructure with a fixed voxel size and desired pore size range, a specific set of length-scale ranges needs to be chosen. Once the relationship between the pore size and length scales is established, the microstructure pore size and porosity could be tuned to achieve desired transport properties. Figure 3 illustrates the generated porous phase with different length scales α = 0.02 – 0.08. The pore size increases linearly with increased length scales α, with corresponding average pore sizes 95, 190, and 380 nm for α = 0.02, α = 0.04, and α = 0.08. In the following analysis, α is primarily varied to control and generate structures with the desired pore size. In Figures 3 and 4, the structures have 200^3^ voxels, and the voxel size is 25 nm. The sensitivity test results of the voxel resolution and domain size are listed in Tables S5 and S6 of the Supporting Information. The selected voxel resolution and domain size can give a reliable prediction of microstructural properties.

The effect of other length scales (*l* and *n* in eq 2) on the microstructure is illustrated in Figure 4. For example, the tortuosity factor in the range of 1.5–5 shows a gradual decrease with an increased porosity fraction; the median pore size in the range of 100–240 nm increases linearly with increasing porosity; the specific surface area (SSA) in the range of 9–17 μm^–1^ increases and then decreases with increased porosity and is greatest when the porosity ε = 0.5. As the length scale increases, the pore size and SSA show increasing and decreasing trends, respectively, while as expected the tortuosity is insensitive to length-scale variations. Having established the ability to describe porosity and other electrode features consistent with experimental observations,^6,22^ the detailed effects of the CBD phase and porosity fraction on the battery performance were investigated.

## 4 Results and Discussion

### 4.1 Electrode Structure Design with Varied CBD Phase Volume Fractions

**Figure 5:**
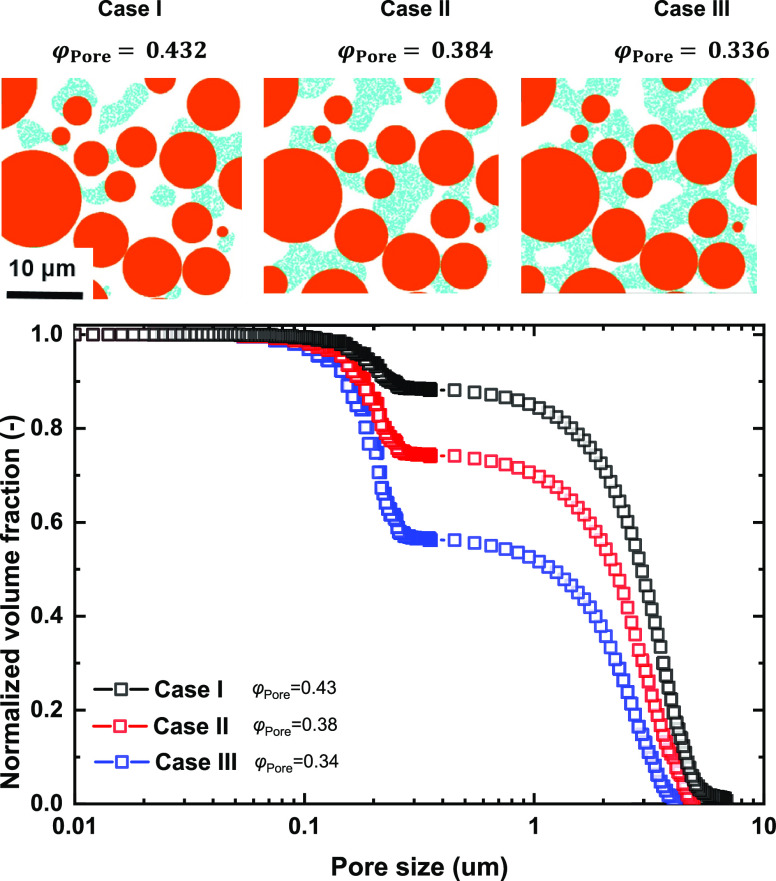
Electrode structure design with varied binder phase and pore phase volume fractions φ_Pore_.

As illustrated in Figure 5, electrode structures with varying CBD phase volume fractions were generated. The structures have 1200^3^ voxels with a voxel size of 25 nm. They contain the particle phase, macropore phase, and microporous CBD phase. In this case, the AM particle skeleton is based on the as-cast NMC622 cathode structure similar to that shown in Figure 1.

The microstructure generation approach readily allows for different volume fractions of macropores and micropores within an electrode to be considered. The volume fraction of macropores φ_Macro_ within an electrode structure was calculated by
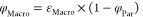
5where φ_Par_ is the volume fraction of the particle phase within an electrode structure, with φ_Par_ = 0.52 for the as-cast structure in Figure 5. ε_Macro_ is defined as the porosity of the macropore structure, i.e., the pure macropore phase porosity without AM particles and the microporous CBD phase. The microporous CBD phase is a combination of micropores and the solid binder phase; therefore, a micropore phase porosity within CBD ε_Micro_ is defined. The volume fraction of micropores φ_Micro_ is a product of microporosity ε_Micro_ and CBD phase volume fraction of an electrode structure, which was calculated by
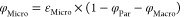
6

By combining eqs 5 and 6, the total volume fraction of the porous phase, i.e., the electrode porosity, φ_Pore_, was calculated as
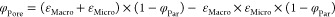
7

The normalized volume fractions of macropores φ_Macro_ and micropores φ_Micro_ were calculated as
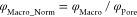
8
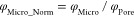
9

As described in Section 2.2, the porosity of the macropore phase ε_Macro_ and micropore phase ε_Micro_ can be controlled via the thresholding random field approach. As shown in Figure 5, with decreasing electrode porosity φ_Pore_, the interparticle CBD phase fraction increases from case I to case III. The properties of the three structures in Figure 5 are listed in Table 1. Figure 5 depicts their bimodal pore size distributions, representing the normalized volume fractions of macropores and micropores. This is the first time that such a bimodal pore size distribution has been achieved via numerical algorithms. Figure 5 shows that the normalized volume fraction of macropores φ_Macro_Norm_ decreases from 0.89 to 0.57 with increasing CBD fraction.

**Table 1:** Volume Fractions of Different Pore Phases within Electrodes

	case I, φ_Pore_ = 0.432	case II, φ_Pore_ = 0.384	case III, φ_Pore_ = 0.336
micropore phase porosity ε_Micro_	0.5	0.5	0.5
macropore phase porosity ε_Macro_	0.8	0.6	0.4
micropore phase volume fraction φ_Micro_	0.048	0.096	0.144
macropore phase volume fraction φ_Macro_	0.384	0.288	0.192

**Figure 6:**
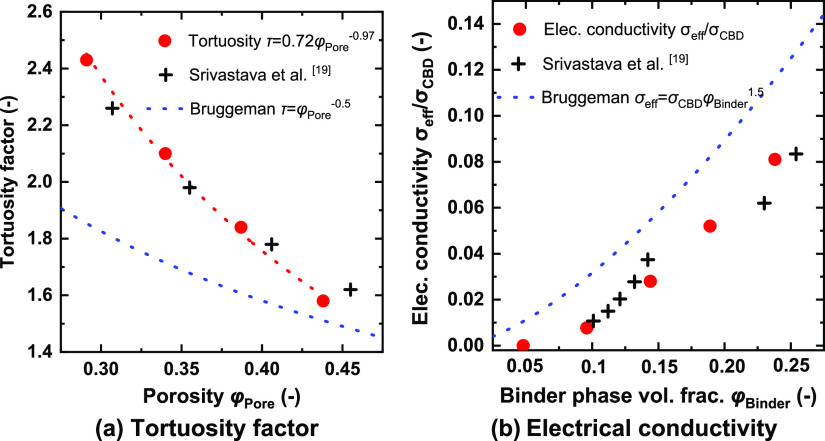
Calculated properties of synthetic electrode structures with varied binder phase and pore phase volume fractions: (a) tortuosity factor and (b) electrical conductivity.

**Figure 7:**
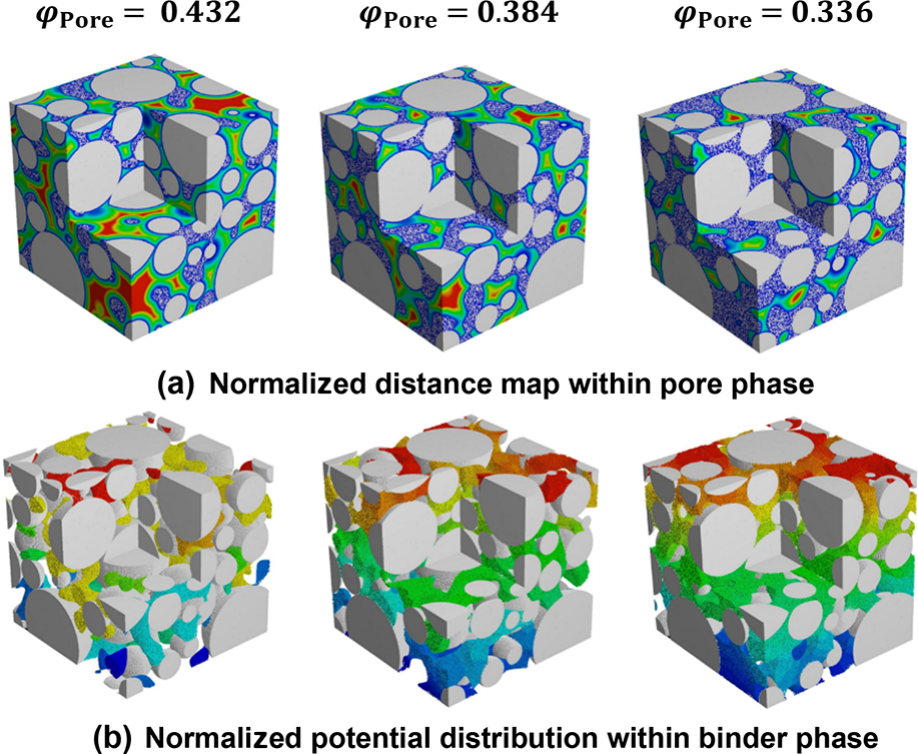
Calculated properties of synthetic electrode structures with varied binder phase and pore phase volume fractions: (a) normalized distance map within the pore phase tortuosity factor and (b) normalized potential distribution within the binder phase.

The electrical properties and tortuosity factors of the generated structures with different total porosities φ_Pore_ are presented in Figure 6. As shown in Figure 6a, the tortuosity varies from 1.5 to 2.4, and the data can be fitted as . The normalized distance map within the porous phase is calculated using a Euclidean distance transform (Figure 7a), showing the decreased macropore size with an increased binder fraction. The predictions agree well with previous research work using a 96% AM weight loading.^19^ The conventional Bruggeman effective medium equation  underpredicts the tortuosity values, which is consistent with previous research.^3^ The resulting electrical conductivity of different structures was calculated by Ohm’s law. As shown in Figure 6b, the predictions from the Bruggeman effective medium equation  are slightly higher than the calculated electrical conductivity when the binder phase volume fraction is 0.05–0.25. A very similar tendency has also been observed in previous work.^19^ When the total porosity is φ_Pore_ = 0.432 (case I in Figure 5), the electrical conductivity is 1.7 × 10^–6^ S/cm, indicating that an electrical percolation network is not established within the electrodes. The corresponding normalized potential distribution is plotted in Figure 7b.

### 4.2 Synthetic Electrode Structures under Varying Calendering Conditions

The previous section sought to describe the as-cast electrode structure. In practice, all electrodes undergo calendering (rolling compression) before use; therefore, in this section, an approach that captures the key features of calendering on the electrode structure was developed.

First, to capture the effect of calendering, AM particle structures with different volume fractions (φ_Par_) were generated using particle packing algorithms (see Table 2). For the electrode structure with known total porosity φ_Pore_, carbon black mass ratio *x* (in wt %), and binder mass ratio *y* (in wt %), the relationship between the total electrode porosity φ_Pore_ and the AM particle volume fraction φ_Par_ is
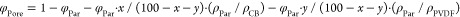
10where the AM particle density ρ_Par_ = 4.7 gcm^–3^, carbon black density ρ_CB_ = 2 gcm^–3^, and binder density ρ_PVDF_ = 1.78 gcm^–3^.^10^

**Table 2:** Volume Fractions of Different Phases within Electrodes under Different Calendering Conditions

	calendered structure I	calendered structure II	calendered structure III
total porosity φ_Pore_	0.39	0.30	0.26
particle phase volume fraction φ_Par_	0.54	0.62	0.65
binder phase volume fraction φ_Binder_	0.07	0.08	0.09

Therefore, the particle phase volume fraction φ_Par_ can be estimated using known composition mass ratios and electrode porosity φ_Pore_:
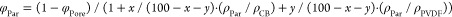
11

The detailed volume fractions of electrode structures under varying calendering conditions were calculated by eq 11 and are listed in Table 2. The carbon black mass ratio is *x* = 2 (in wt %) and the binder mass ratio is *y* = 3 (in wt %).^32^ Experimental porosimetry data by mercury intrusion (Supporting Information, Figure S1) were used to extract electrode structure properties such as macropore volume fraction φ_Macro_, micropore volume fraction φ_Micro_, and corresponding average pore size (*D*_Micro_50_ and *D*_Macro_50_), as listed in the Supporting Information (Table S1). By using these parameters, the synthetic electrode structures under different calendering conditions were obtained.

**Figure 8:**
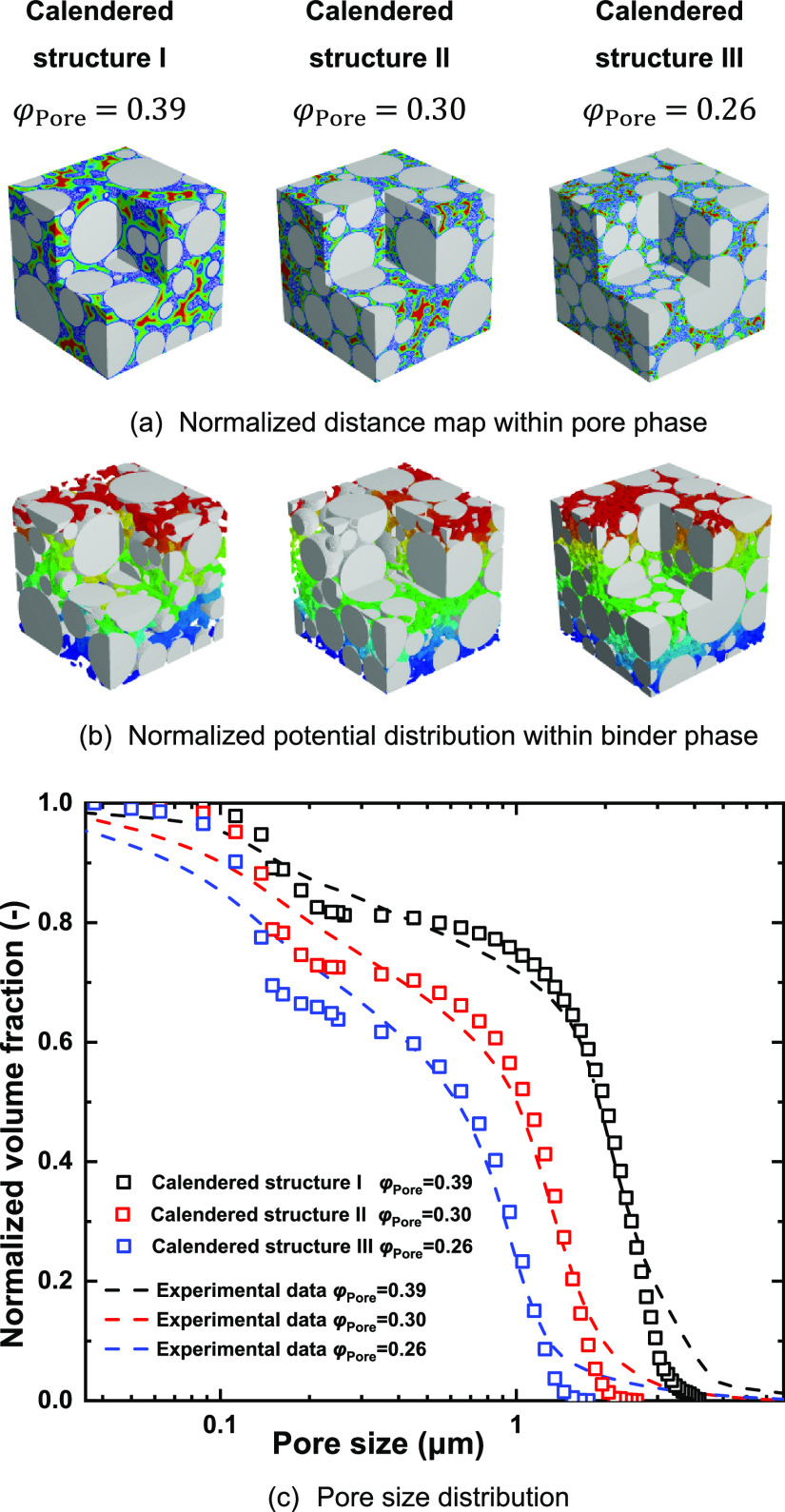
Synthetic electrode structures under varying calendering conditions and corresponding pore size distribution: (a) normalized distance map within the pore phase, (b) normalized potential distribution within the binder phase, and (c) pore size distribution. The experimental data is taken from ref (32).

Figure 8 shows the corresponding normalized distance map, potential distribution, and cumulative volume fraction of pore size distribution of synthetic electrode structures. As expected, the pore size decreases with increased calendering. The cumulative bimodal pore size distribution of the synthetic electrode structures was compared with the mercury intrusion test results under the same calendering conditions,^32^ with excellent agreement. There is a slight discrepancy when the pore size is smaller than 0.3 μm and likely arises because a 25 nm voxel size and an average micropore size (>100 nm) are used to minimize the computation time. In future, the effect of pore size distribution variations at an increased spatial resolution will be explored. The CBD-only domains with high voxel resolutions can be generated to tune the pore size distributions and microstructural properties.

**Figure 9:**
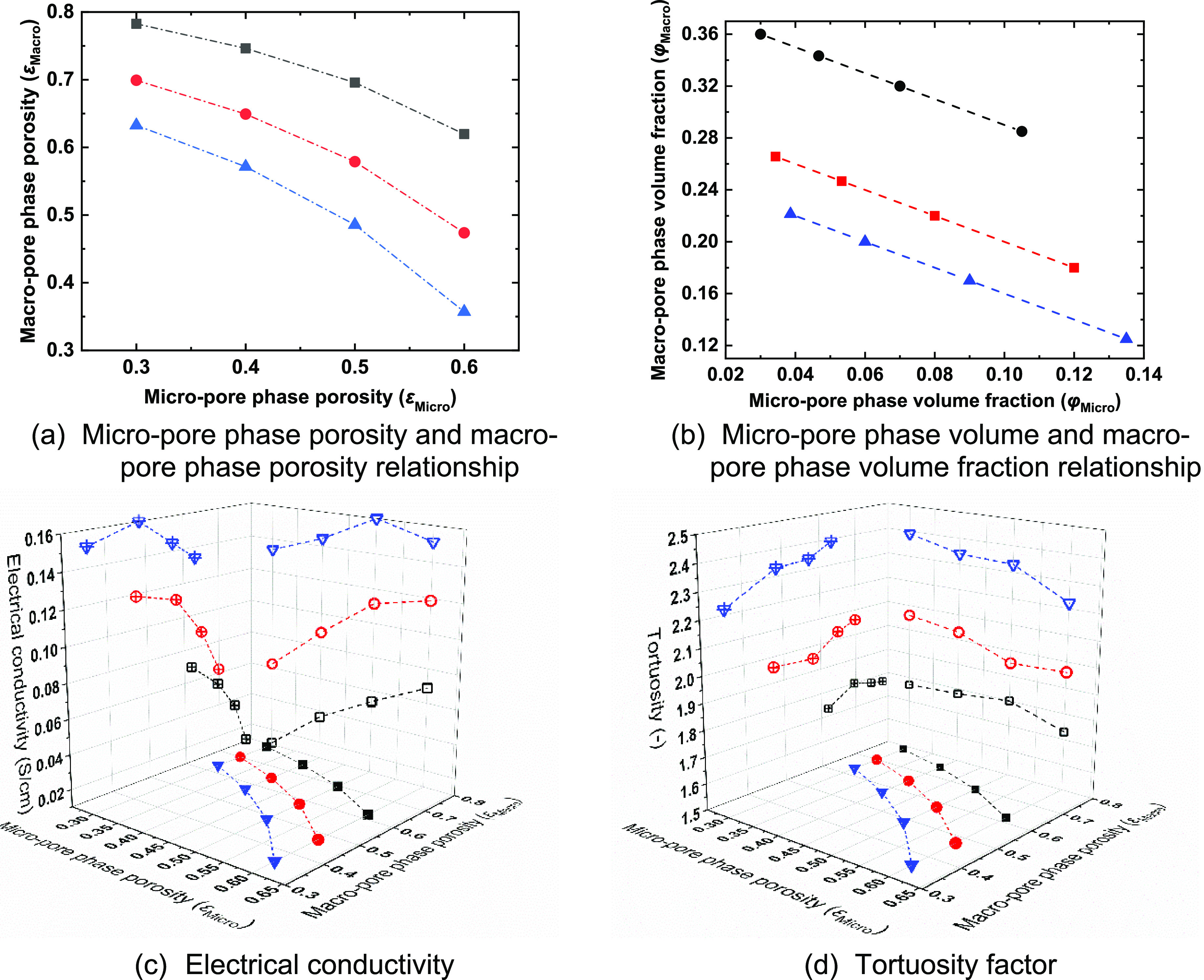
Structure properties under different calendering conditions and the calculated electrical conductivity and tortuosity. (a, b) Porosity relationship. (c, d) Electrical conductivity and tortuosity factor. The black, red, and blue curves represent the calendered structure I φ_Pore_ = 0.39, calendered structure II φ_Pore_ = 0.3, and calendered structure III φ_Pore_ = 0.26, respectively.

For a structure with a total porosity φ_Pore_, the relationship of the macropore phase and micropore phase was established using eq 7. The micropore phase porosity ε_Micro_ and macropore phase porosity ε_Macro_ of different calendered structures were calculated and are presented in Figure 9a,b. Accordingly, the volume fractions of macropores φ_Macro_ and micropores φ_Micro_ were calculated using eqs 5 and 6, and the results are shown in Figure 9b. The detailed structure properties of these synthetic structures are listed in the Supporting Information (Tables S2–S4).

Figure 9c,d summarizes the variation of electrical conductivity and tortuosity as a function of the micropore phase ε_Micro_ and macropore phase porosity ε_Macro_. For all three structures with a total porosity φ_Pore_, the electrical conductivity decreases as the micropore phase porosity ε_Micro_ decreases and macropore phase porosity ε_Macro_ increases. Tortuosity is mainly affected by the total porosity of the three calendered structures. For a given total porosity φ_Pore_, tortuosity slightly decreases with increasing microporosity φ_Micro_. In previous work, electrical conductivity and tortuosity were typically related to the average electrode porosity, for example, using Bruggeman’s model.^3^ By considering only average porosity, this approach is limited as it does not consider the relative contributions of macro- and microporosity; however, the results in Figure 9 demonstrate that the effects of multiscale pores are substantial. The importance of the multiscale pore size distribution is further evidenced by examination of the effect of calendering. For electrodes calendered to the same overall porosity with a controlled CBD phase volume fraction φ_Binder_, an increased CBD phase microporosity φ_Micro_ improves electrode transport properties, e.g., for structure I, the largest electrical conductivity 0.07 S/cm with the lowest tortuosity 1.73 was achieved when ε_Micro_ = 0.6 (Figure 9c). A counterintuitive effect is observed that with an increased CBD phase microporosity φ_Micro_, the effective CBD phase conductivity decreases, while the total electrode electrical conductivity increases. This is possibly due to better connectivity of the porous CBD phase spanning multiple AM particles. The results in Figure 9 also demonstrate that increased calendering can enhance electron transport properties but inhibit ionic transport properties. For example, at a constant microporosity of the CBD phase ε_Micro_ = 0.5, changing the total porosity φ_Pore_ from 0.39 to 0.26 results in an increased electrical conductivity from 0.05 to 0.16 S/cm, while tortuosity increases from 1.8 to 2.3 (Figure 9c,d). Overall, the results illustrate a significant influence of the CBD phase microstructure despite its relatively low fraction.

An electrochemical finite element model was used to evaluate the impact of the microporous CBD phase with varied microporosity ε_Micro_(0.3–0.6) under different calendering conditions listed in Table 2. The methodology and theoretical framework can be found in the Supporting Information. The approach is essentially standard for the field, apart from the richer description of porosity and interlinked properties, as described in the previous sections. Structures I–III were incorporated into the model and simulations used to explore the discharge behavior at rates of 0.1, 1, and 3C. To incorporate the CBD into the microscale simulations, it was necessary that the details of the micropore phase were homogenized to the porosity and tortuosity measured previously (see Figure 4). Microporosities ε_Micro_ of 0.3, 0.4, 0.5, and 0.6 were prescribed to the various microstructures. The corresponding effective electrical conductivity of the microporous CBD phase is 225–752 S/m, as calculated in Section 2.2.

**Figure 10:**
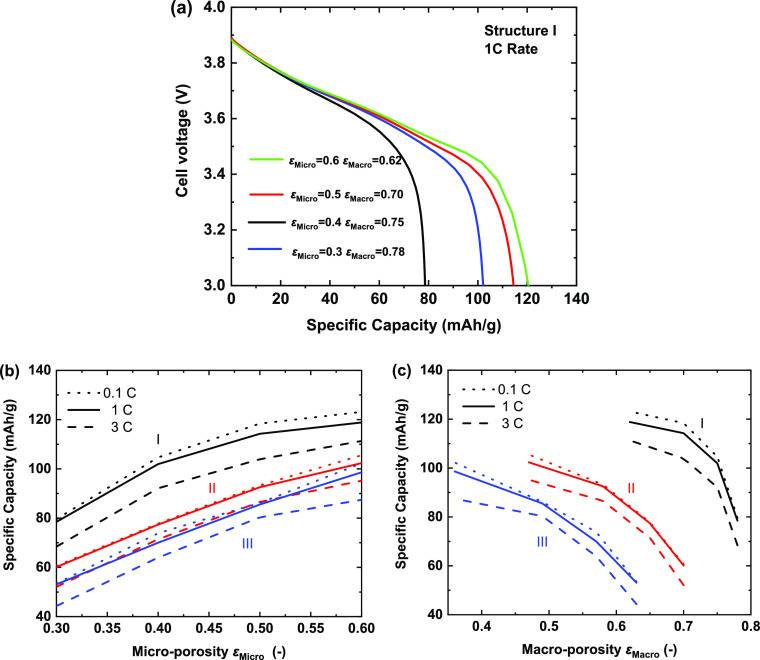
(a) Voltage response for structure I as a function of microporosity (ε_Micro_) at a rate of 1C. (b, c) Specific capacity (at 3 V) as a function of microporosity and macroporosity for 0.1, 1, and 3C discharges and structures I–III.

In broad terms, the electrode capacity is significantly affected by the microporosity ε_Micro_ of the CBD phase and macroporosity ε_Macro_ of the whole electrode structure. Figure 10a shows that for a constant electrode macrostructure (structure I), variations in the microporosity fraction can have a surprisingly strong effect on achievable capacity. At 1C, all the achievable capacities are significantly below theoretical due to the high macroporosity and tortuosity, but under these conditions, increases in microporosity ε_Micro_ from 0.3 to 0.6 can increase capacity from 80 to 120 mAh/g. Figure 10b plots the specific capacity as a function of microporosity ε_Micro_ for all three structures and discharge rates from 0.1 to 1C. All the electrode structures (I–III) with low microporosity show low specific capacity: when the microporosity ε_Micro_ = 0.3, the specific capacities are 50–80 mAh/g at a 1C discharge rate. This is a direct consequence of high tortuosity (τ_Micro_ = 4.5 when ε_Micro_ = 0.3) in the micropores of the CBD phase. This can also be evidenced by the transport property results in Figure 9, e.g., for structure I, a lowest electrical conductivity of 0.01 S/cm and a highest tortuosity of 1.77 are observed when ε_Micro_ = 0.3. A lower microporosity also provides a reduced surface area for electrochemical reactions at the particle/electrolyte interface. When the microporosity increases to ε_Micro_ = 0.6, the specific capacity is 1.5 to 2 times higher. As shown in Figure 9a, with increased microporosity ε_Micro_, the macroporosity ε_Macro_ decreases. Figure 10c describes the effect of macroporosity ε_Macro_ on the achievable capacities. As expected, the specific capacity decreases with increased macroporosity for all the electrode structures (I–III).

**Figure 11:**
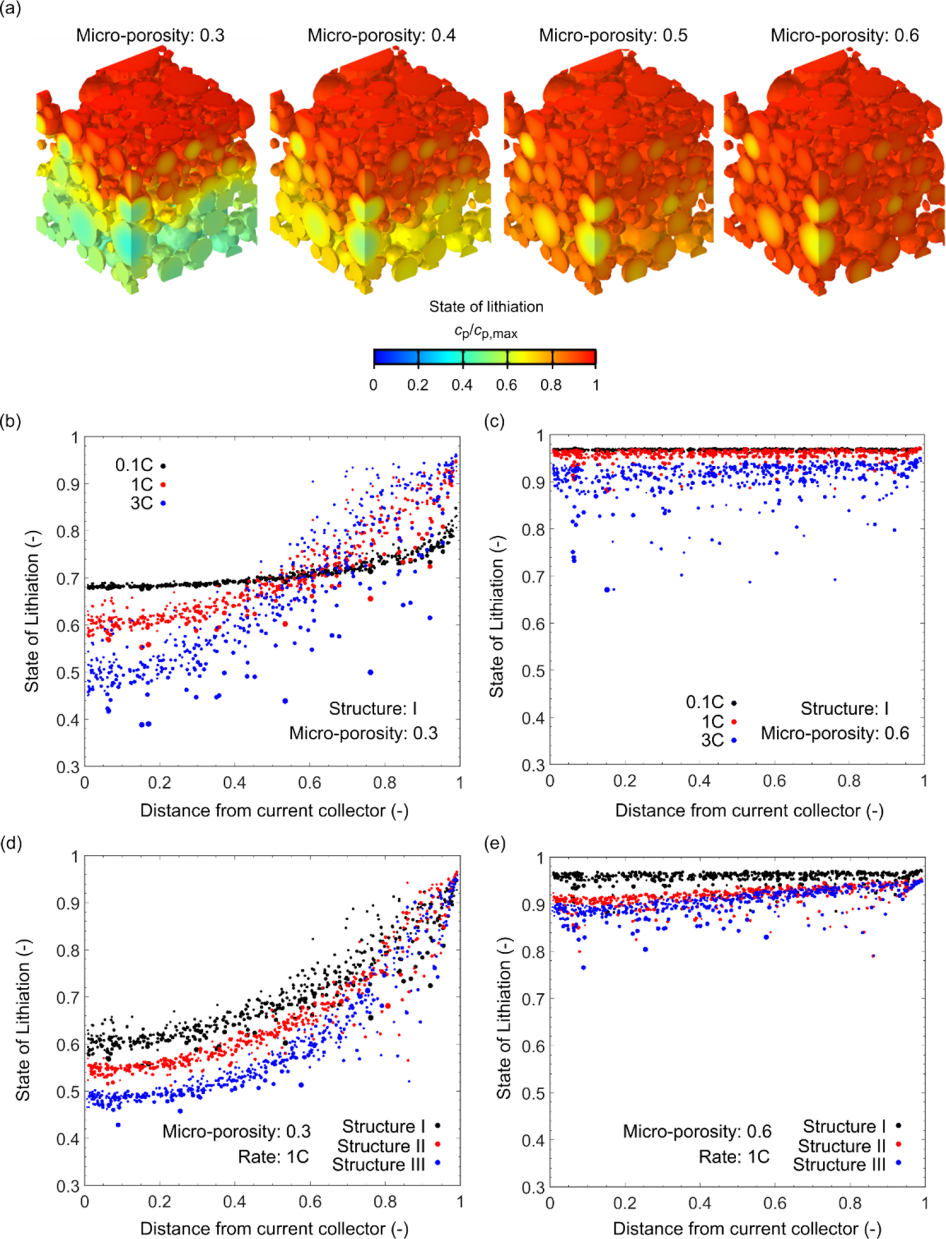
(a) SoL profiles as a function of microporosity ε_Micro_ for structure I at a discharge rate of 1C. (b, c) SoL profiles as a function of distance from the current collector for structure I at 0.1, 1, and 3C discharges for (b) 0.3 and (c) 0.6 microporosities. (d, e) SoL profiles as a function of distance from the current collector for structure I at 1C, for structures I–III at (d) 0.3 and (e) 0.6 microporosities.

State-of-lithiation (SoL) profiles provide further insight into the electrode performance as a function of microporosity ε_Micro_, as exemplified in Figure 11a, which shows how regions of high SoL gradually penetrate through the electrode thickness as the microporosity ε_Micro_ increased. In this case, the separator is at the top of the electrode and the current collector at the base. It is clear that there is a size dependence in SoL, as illustrated in Figure S3 of the Supporting Information, similar to that shown in the work of Ferraro et al.^33^ Large particles have lower lithium concentrations than their small counterparts at a given location within an electrode. As in Ferraro et al.,^33^ we see a scatter in SoL, even for small, similarly sized particles at the same location within the electrode, for which they offered various hypotheses. We now demonstrate that CBD morphology and rate/kinetics considerations directly influence the level of this scatter. Figure 11b,c shows the SoL at the end of discharge as a function of distance from the current collector for structure I and for varying porosity and discharge rates. In general, the discharge rate has a significant influence on the SoL distribution. Electrodes discharged at high rates (3C discharges in this work) experience significant gradients in AM utilization, and this can be correlated with sluggish diffusion in both the active particles (micron length scale) and particularly within the electrolyte (10’s micron scale).^29^ As implied in Figure 10a, increasing microporosity ε_Micro_ within the CBD phase has a significant effect on reducing SoL gradient and improves overall AM utilization. This is also evident in the SoL gradients at 1C discharge in Figure 11d,e. Thus, the influence of porosity is multiscale, and both macropores and micropores within the CBD phase must be taken into consideration when designing an optimized electrode microstructure.

### 4.3 Discussion

In this work, we proposed a new numerical design framework of CBD microstructures. By using this approach, the effects of CBD microstructures on the battery performance were systematically investigated. For the first time, CBD microstructures with bimodal pore size distributions were precisely controlled via numerical algorithms, and the results were quantitatively validated by porosimetry experiments. As shown in Figure 2, to reduce computational resources, the CBD can be homogenized by just considering the macropore phase (Figure 2b). Further, the microporous CBD can be reconstructed with micropores at a submicron scale (Figure 2c). This approach opens up the possibility to couple with AM particle scale modeling to investigate the effect of calendering conditions. In DEM simulations of the calendering process, the porous CBD microstructure evolution can hardly be modeled and validated by experiments.^34^ By using the proposed approach, the CBD phase can be easily reconstructed between AM particles under different calendering conditions.

The proposed numerical framework is used to decouple the effect of granular microstructures, calendering behavior, and CBD microporosity. In practice, previous results show that the battery performance is sensitive to the heterogeneity of electrode microstructures including particle size distribution and CBD morphology.^3,19^ In this work, heterogeneous CBD microstructures were generated by the thresholding random field approach. It was found that with a controlled binder phase volume fraction, the pore size distribution can significantly affect the battery performance. The results also show coupling mechanisms of CBD microstructures and calendering levels on the battery performance. As have been illustrated in Figures 1 and 8, the micrometer-scale macropore size varies during calendering. With increased calendering levels, the pore phase volume fraction φ_Pore_ decreases. As shown in Figure 9, this can enhance electron transport properties but will also inhibit ionic transport properties. Figure 10 shows that both calendering conditions and varied CBD microporosity affect the battery performance. The SoL profiles at individual particle scales are illustrated in Figure 11. A wide variation of AM utilization can be observed that is affected by particle size and position, as well as the CBD morphology. Similar findings have been reported in our previous work and Ferraro et al.^33,34^ Apart from this, it is clear that increased CBD microporosity ε_Micro_ can improve the overall AM particle utilization.

This study has also highlighted several issues for future research. In this work, the properties of carbon black filler particles are described by the intrinsic electrical conductivity of the solid binder phase. With the assistance of advanced experimental characterization techniques, the effect of carbon black filler distributions could be measured and incorporated into this numerical framework to calculate the effective properties of the microporous CBD. In reality, manufacturing processes such as mixing, drying, and calendering have an enormous impact on spatial distributions of the CBD phase.^2^ Emerging manufacturing processes need to be considered to precisely tailor the microporous CBD phase, e.g., dry powder deposition and additive manufacturing.^7^

## 5 Conclusions

The intricate relationship between the properties of the microporous CBD phase and subsequent battery performance has been investigated using a thresholding random field approach that can capture the complex pore size characteristic of real LIB electrodes. The resulting bimodal pore size distributions have been validated by a combination of qualitative and quantitative approaches using microscopy and porosimetry.

The effective transport properties of NMC-based electrodes were calculated for two different cases: first, the NMC volume fraction φ_Par_ and micropore phase porosity ε_Micro_ were fixed and the binder phase volume fraction φ_Binder_ was varied; and second, the volume fraction of the NMC volume fraction φ_Par_ was varied under three calendering conditions. The resulting relationships between the NMC volume fraction φ_Par_, CBD phase microstructure, and electrode transport properties were investigated in detail. With a controlled CBD fraction, improvements in electrode transport properties were provided by increases in microporosity ε_Micro_. Both macropores and the microporous CBD phase were shown to play an important role in electrode dynamics and achievable energy storage response. For example, an increase in microporosity ε_Micro_ from 0.3 to 0.6 was shown to increase specific capacity by 50 to 100% under certain conditions.

The generic approach described here provides an additional degree of structural freedom (e.g., porosity at different length scales) that can be exploited to tune the overall electrode response, for example, to maintain useful overall capacity in thicker electrodes that otherwise would show reduced AM utilization and overall capacity loss. Future work will include the development of experimental approaches that seek to decouple micro- and macroporosity distributions in practice, which could serve to both help demystify the complex effects of the microporous CBD phase on the battery performance and provide greater design freedom.

## Supporting Information Available

Experimental data analysis for structure modeling (Figure S1 and Tables S1–S4), sensitivity test of voxel resolution and domain size (Tables S5 and S6), and electrochemical simulation (Figures S2 and S3 and Tables S7–S10) (PDF)

## Nomenclature

*C*: covariance function, –
*D*_Micro_50_, *D*_Macro_50_: average pore size, nm
*l*: length scale of the generated microstructure, –
*N*: total number of voxels, –
*n*: length scale of the generated microstructure, –
*T*: Gaussian random fields, –
*W*: normally distributed three-dimensional array, –
*x*: carbon black mass ratio, wt %
*y*: binder mass ratio, wt %
**Greek symbols**
α: length scale of the generated microstructure, –
γ: spectral density, –
ε: porosity, –
ε_Micro_: micropore phase porosity (microporosity within the CBD phase), –
ε_Macro_: macropore phase porosity (macroporosity), –
ρ_Par_: particle density, m^3^ kg^–1^
ρ_CB_: carbon black density, m^3^ kg^–1^
ρ_PVDF_: binder density, m^3^ kg^–1^
σ_0_: intrinsic electrical conductivity, S m^–1^
σ_eff_: effective electrical conductivity, S m^–1^
τ_Pore_: tortuosity factor of porous phase, –
Φ: binary description of the generated microstructure, –
φ: volume fraction, –
φ_Par_: particle phase volume fraction within the whole structure, –
φ_Binder_: binder phase volume fraction within the whole structure, –
φ_Pore_: pore phase volume fraction within the whole structure (total porosity), –
φ_Micro_: micropore phase volume fraction within the whole structure, –
φ_Macro_: macropore phase volume fraction within the whole structure, –
φ_Micro_Norm_: normalized micropore phase volume fraction within the whole structure, –
φ_Macro_Norm_: normalized macropore phase volume fraction within the whole structure, –
