# Supplementary material for: Numerical Design of Microporous Carbon Binder Domains Phase in Composite Cathodes for Lithium-Ion Batteries
Source: ACS Appl Mater Interfaces. 2023 May 31;15(23):27809–20. doi: 10.1021/acsami.3c00998 (PMC10273235; doi:10.1021/acsami.3c00998)
Supplement: Supplementary file 1 — am3c00998_si_001.pdf [file am3c00998_si_001.pdf]

# Supporting Information

# Numerical Design of Microporous Carbon Binder Domains in Composite Cathodes for Lithium-Ion Batteries

*Ruihuan Ge[1,5], Adam M. Boyce[2,4], Yige Sun[3,5], Paul R. Shearing[2,5], Patrick S. Grant[3,5], Denis Cumming[1,5]*, Rachel M. Smith[1,5]**

*[1]Department of Chemical and Biological Engineering, The University of Sheffield, Sheffield, S10 2TN, UK*

*[2]Electrochemical Innovation Lab, Department of Chemical Engineering, University College London, London, WC1E 7JE, UK*

*[3]Department of Materials, University of Oxford, Oxford OX1 3PH, UK*

*[4]School of Mechanical and Materials Engineering, University College Dublin, Dublin 4, Ireland*

*[5]The Faraday Institution, Quad One, Harwell Science and Innovation Campus, Didcot, OX11 0RA, UK*

* Corresponding author: d.cumming@sheffield.ac.uk; rachel.smith@sheffield.ac.uk

## 1. Experimental data analysis for structure modelling

The experimental data of bimodal pore size distribution of three calendered structures in literature[1] are shown in Figure S1. From this figure, we can distinguish the micro-pore phase and macro-pore phase. The structure properties can be calculated using Figure S1. The properties used for generating the micro-porous CBD phase are listed in Table S1.

For each structure with a certain porosity, the microstructures of micro-porous CBD phase can be changed by varying the porosity of micro-pore and macro-pore phase. Detailed properties of each structure are listed in Tables S2-S4.

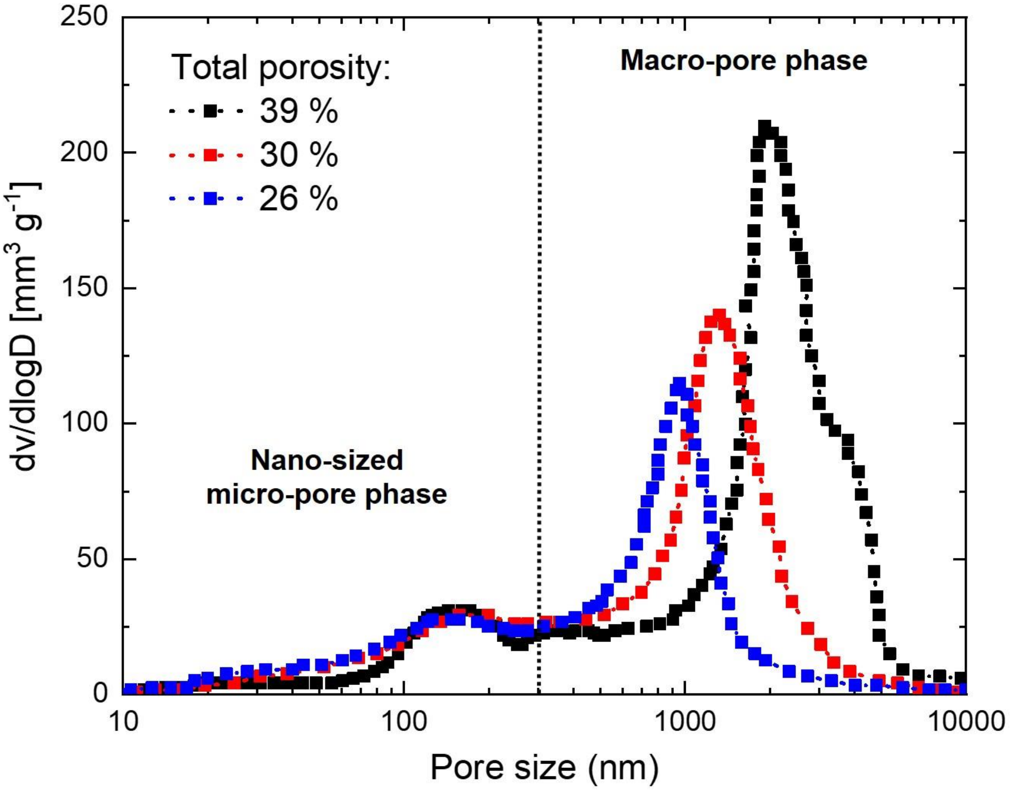


**Figure S1.** Bimodal pore size distribution of electrode microstructures from mercury intrusion experiments[1].

**Table S1.** Electrode structure properties extracted from experimental data.

| | $\varphi_{\text{Pore}} = 0.39$ | $\varphi_{\text{Pore}} = 0.30$ | $\varphi_{\text{Pore}} = 0.26$ |
|---|---|---|---|
| $\varphi_{\text{Micro\_Norm}}$ | 0.18 | 0.27 | 0.35 |
| $\varphi_{\text{Macro\_Norm}}$ | 0.82 | 0.73 | 0.65 |
| $D_{\text{Micro\_50}}$ | 140 nm | 130 nm | 115 nm |
| $D_{\text{Macro\_50}}$ | 2100 nm | 1238 nm | 880 nm |

**Table S2.** Properties of calendered structure I (Total porosity $\varphi_{\mathrm{Pore}} = 0.39$ Particle phase volume fraction $\varphi_{\mathrm{Par}} = 0.54$).

| | | | | |
|---|---|---|---|---|
| Micro-pore phase porosity $\varepsilon_{\mathrm{Micro}}$ | 0.3 | 0.4 | 0.5 | 0.6 |
| Macro-pore phase porosity $\varepsilon_{\mathrm{Macro}}$ | 0.78 | 0.75 | 0.70 | 0.62 |
| Macro-pore phase volume fraction $\varphi_{\mathrm{Macro}}$ | 0.36 | 0.34 | 0.32 | 0.29 |
| Micro-pore phase volume fraction $\varphi_{\mathrm{Micro}}$ | 0.03 | 0.047 | 0.07 | 0.11 |
| Normalised Macro-pore phase volume fraction $\varphi_{\mathrm{Macro\_Norm}}$ | 0.92 | 0.88 | 0.82 | 0.73 |
| Normalised Micro-pore phase volume fraction $\varphi_{\mathrm{Micro\_Norm}}$ | 0.08 | 0.12 | 0.18 | 0.27 |

**Table S3.** Properties of calendered structure II (Total porosity $\varphi_{\mathrm{Pore}} = 0.30$ Particle phase volume fraction $\varphi_{\mathrm{Par}} = 0.62$).

| | | | | |
|---|---|---|---|---|
| Micro-pore phase porosity $\varepsilon_{\mathrm{Micro}}$ | 0.3 | 0.4 | 0.5 | 0.6 |
| Macro-pore phase porosity $\varepsilon_{\mathrm{Macro}}$ | 0.70 | 0.65 | 0.58 | 0.47 |
| Macro-pore phase volume fraction $\varphi_{\mathrm{Macro}}$ | 0.27 | 0.25 | 0.22 | 0.18 |
| Micro-pore phase volume fraction $\varphi_{\mathrm{Micro}}$ | 0.034 | 0.053 | 0.08 | 0.12 |
| Normalised macro-pore phase volume fraction $\varphi_{\mathrm{Macro\_Norm}}$ | 0.88 | 0.82 | 0.73 | 0.6 |
| Normalised micro-pore phase volume fraction $\varphi_{\mathrm{Micro\_Norm}}$ | 0.12 | 0.18 | 0.27 | 0.4 |

**Table S4.** Properties of calendered structure III (Total porosity $\varphi_{\mathrm{Pore}} = 0.26$ Particle phase volume fraction $\varphi_{\mathrm{Par}} = 0.65$).

| | | | | |
|---|---|---|---|---|
| Micro-pore phase porosity $\varepsilon_{\mathrm{Micro}}$ | 0.3 | 0.4 | 0.5 | 0.6 |
| Macro-pore phase porosity $\varepsilon_{\mathrm{Macro}}$ | 0.63 | 0.57 | 0.49 | 0.36 |
| Macro-pore phase volume fraction $\varphi_{\mathrm{Macro}}$ | 0.22 | 0.2 | 0.17 | 0.13 |
| Micro-pore phase volume fraction $\varphi_{\mathrm{Micro}}$ | 0.039 | 0.06 | 0.09 | 0.14 |
| Normalised macro-pore phase volume fraction $\varphi_{\mathrm{Macro\_Norm}}$ | 0.85 | 0.77 | 0.65 | 0.52 |
| Normalised micro-pore phase volume fraction $\varphi_{\mathrm{Micro\_Norm}}$ | 0.15 | 0.23 | 0.35 | 0.48 |

## 2. Sensitivity test of voxel resolution and domain size

The sensitivity test results of voxel resolution and domain size are listed in Tables S5-S6. Five voxel resolutions were tested for a 50 μm volume (Table S5). The effect of domain size (1.25-20 μm) was tested (Table S6).

**Table S5.** The effect of voxel resolution.

| | 6.25 nm | 12.5 nm | 25 nm | 50 nm | 100 nm | 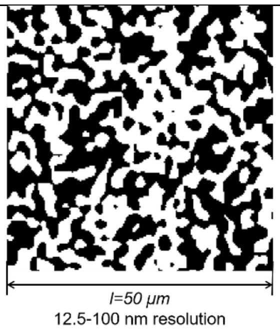 |
|---|---|---|---|---|---|---|
| Tortuosity $\tau$ (-) | 1.83 | 1.88 | 1.98 | 2.24 | 2.85 | |
| Pore size *D50* (nm) | 193.23 | 191.09 | 190.34 | 184.32 | 156.16 | |
| Specific surface area *SSA* ($\mu m^{-1}$) | 11.7 | 11.7 | 11.7 | 11.5 | 10.7 | |

**Table S6.** The effect of domain size.

| | *l*=800 | *l*=400 | *l*=200 | *l*=100 | *l*=50 | 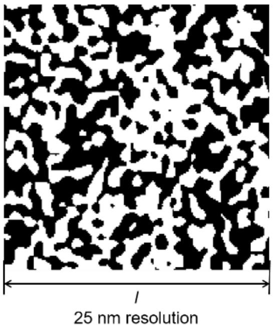 |
|---|---|---|---|---|---|---|
| Tortuosity $\tau$ (-) | 1.99 | 2 | 1.98 | 1.98 | 1.98 | |
| Pore size *D50* (nm) | 190.36 | 190.7 | 190.34 | 194.5 | 193.7 | |
| Specific surface area *SSA* ($\mu m^{-1}$) | 11.7 | 11.7 | 11.7 | 11.7 | 11.7 | |

## 3. Electrochemical simulation

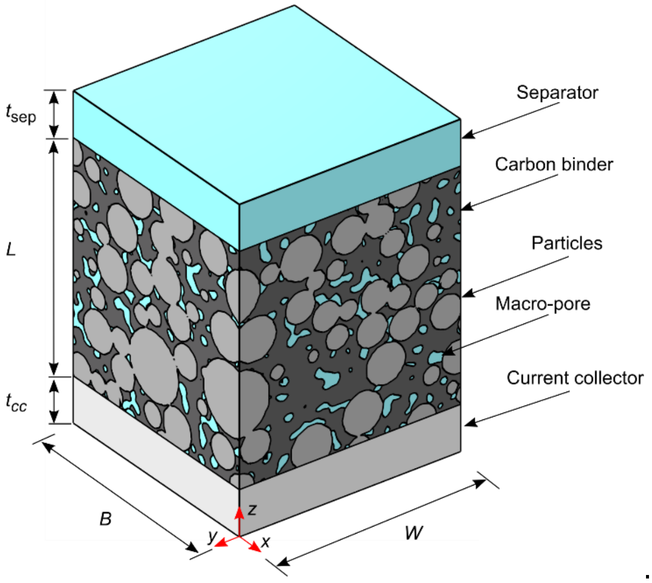


**Figure S2.** Representative electrode geometry for electrochemical simulations. $L$=$B$=W=50 um, $t_{sep}$= $t_{cc}$ =10 um.

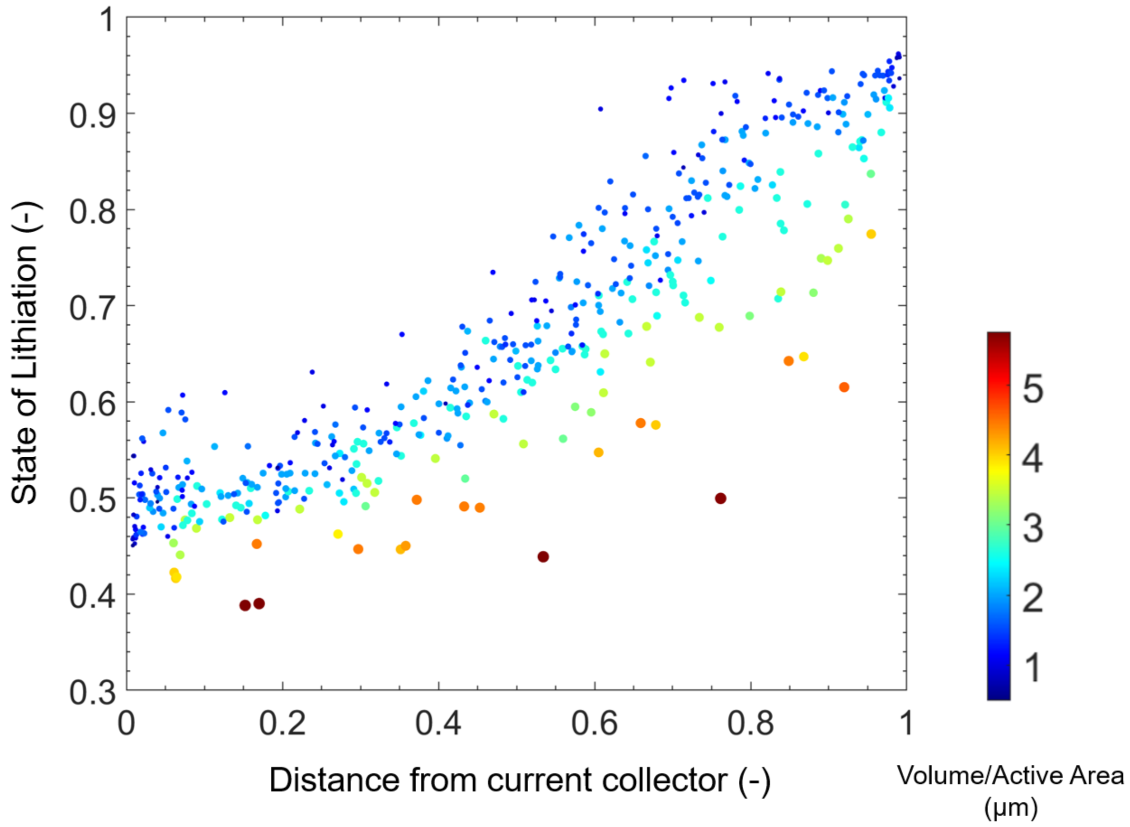


**Figure S3.** State-of-lithiation profile as function of distance from current collector for Structure I, micro-porosity of 0.3 and a C-rate of 3C; the size and colour of markers illustrates the particle size (volume/surface area).

**Table S7.** Model equations for each domain in the 3D model.

| Phase | Equation | Description | Number |
|---|---|---|---|
| Particles | $\boldsymbol{J}_{\mathrm{p}} = -D_{\mathrm{p}} \nabla c_{\mathrm{p}}$ | Fick's first law | (S1) |
| | $\frac{\partial c_{\mathrm{p}}}{\partial t} + \nabla \cdot \boldsymbol{J}_{\mathrm{p}} = 0$ | Fick's second law | (S2) |
| | $\nabla \cdot \boldsymbol{i}_{\mathrm{p}} = 0$ | Charge conservation | (S3) |
| | $\boldsymbol{i}_{\mathrm{p}} = -K_{\mathrm{p}} \nabla \phi_{\mathrm{p}}$ | Ohm's Law | (S4) |
| Electrolyte | $\boldsymbol{J}_{\mathrm{e}} = -D_{\mathrm{e}} \nabla c_{\mathrm{e}} + \frac{\boldsymbol{i}_{\mathrm{e}} t_{+}}{F}$ | Diffusive and migration related species flux | (S5) |
| | $\frac{\partial c_{\mathrm{e}}}{\partial t} + \nabla \cdot \boldsymbol{J}_{\mathrm{e}} = 0$ | Fick's second law | (S6) |
| | $\nabla \cdot \boldsymbol{i}_{\mathrm{e}} = 0$ | Charge conservation | (S7) |
| | $\boldsymbol{i}_{e} = (-K_{\mathrm{e}} \nabla \phi_{\mathrm{e}}) + \frac{2K_{\mathrm{e}} RT}{F} \left( 1 + \frac{\partial \ln f}{\partial \ln c_{\mathrm{e}}} \right) (1 - t_{+}) \nabla \ln c_{\mathrm{e}}$ | Electrolyte conduction-related flux | (S8) |
| Particle-Electrolyte interface | $i_{\mathrm{BV}} = i_0 \left( \exp\left( \frac{\alpha_{\mathrm{a}} F \eta}{RT} \right) - \exp\left( -\frac{\alpha_{\mathrm{b}} F \eta}{RT} \right) \right)$ | Butler-Volmer type equation | (S9) |
| | $i_0 = F (k_{\mathrm{b}})^{\alpha_{\mathrm{a}}} (k_{\mathrm{a}})^{\alpha_{\mathrm{b}}} (c_{\mathrm{p,max}} - c_{\mathrm{p}})^{\alpha_{\mathrm{a}}} (c_{\mathrm{p}})^{\alpha_{\mathrm{b}}} \left( \frac{c_{\mathrm{e}}}{c_{\mathrm{e,ref}}} \right)^{\alpha_{\mathrm{a}}}$ | Local exchange current density | (S10) |
| | $\eta = \phi_{\mathrm{p}} - \phi_{\mathrm{e}} - U$ | Overpotential | (S11) |
| CBD | $\nabla \cdot \boldsymbol{i}_{\mathrm{c}} = 0$ | Charge conservation | (S12) |
| | $\boldsymbol{i}_{\mathrm{c}} = -K_{\mathrm{c}} \nabla \phi_{\mathrm{c}}$ | Ohm's Law | (S13) |

**Table S8.** Boundary conditions. Note that all external domain surfaces normal to the *x-z* plane and *y-z* plane are assumed to be insulating to active material-, CBD-, and electrolyte-related current densities, as well as species fluxes. An initial active material concentration, $c_{p0}$, is prescribed, whilst the electrolyte initial concentration is given by $c_{e0}$.

| Interface | Equation | Location | Number |
|---|---|---|---|
| Current collector/Electrode | $\boldsymbol{i}_{c} \cdot \boldsymbol{n}_{cc} = i_0$ ; $i_0$= SoL*$c_{p,max}FV_p/3600A_s$ | $z$=0 | (S14) |
| | $SoL = SoL_{max} - SoL_{min}$ | | (S15) |
| | $\boldsymbol{u} \cdot \boldsymbol{n}_{cc} = i_0$ | | (S16) |
| Separator | $\boldsymbol{J}_{e} \cdot \boldsymbol{n}_{el} = i_{in} / F$ | $z=t_{cc}+L+t_{sep}$ | (S17) |
| | $\boldsymbol{i}_{e} \cdot \boldsymbol{n}_{el} = -i_{in}$ | | (S18) |
| | $\phi_p$=0 | | (S19) |
| Separator/heterogeneous electrode | $\boldsymbol{i}_{c} \cdot \boldsymbol{n}_{se} = 0$ | $z=L+t_{cc}$ | (S20) |
| | $\boldsymbol{i}_{p} \cdot \boldsymbol{n}_{se} = 0$ | | (S21) |
| Active material | $\boldsymbol{J}_{e} \cdot \boldsymbol{n}_{pe} = -i_{BV} / F$ ; $\boldsymbol{J}_{s} \cdot \boldsymbol{n}_{pe} = -i_{BV} / F$ | Particle surfaces | (S22) |
| | $\boldsymbol{i}_{e} \cdot \boldsymbol{n}_{pe} = -i_{BV}$ ; $\boldsymbol{i}_{p} \cdot \boldsymbol{n}_{pe} = i_{BV}$ | | (S23) |

**Table S9.** Material properties and model parameters in electrochemical simulation.

| Parameter | Unit | Value | Source |
|---|---|---|---|
| $t_+$ | 1 | 0.37 | Valøen et al.[2] |
| $\frac{\partial \ln f}{\partial \ln c_e}$ | 1 | 0.43 | Valøen et al.[2] |
| $D_e$ | $m^2\ s^{-1}$ | f($c_e$) | Valøen et al.[2] |
| $K_e$ | $S\ m^{-1}$ | f($c_e$) | Valøen et al.[2] |
| $c_{e0}$ | $mol\ m^{-3}$ | 1000 | - |
| $D_p$ | $m^2\ s^{-1}$ | f($c_p/c_{pmax}$) | Noh et al.[3] |
| $c_{p,max}$ | $mol\ m^{-3}$ | 48700 | Xu et al.[4] |
| $K_p$ | $S\ m^{-1}$ | $1.6\times10^{-4}$ | Noh et al.[3] |
| $c_{p0}$ | $mol\ m^{-3}$ | 500 | - |
| $V_p$ | $m^3$ | $6.54\times10^{-14}$ | - |
| $K_c$ | $S\ m^{-1}$ | 225-752 | - |
| $\alpha_a$ | 1 | 0.5 | - |
| $\alpha_b$ | 1 | 0.5 | - |
| $k_a$ | $m\ s^{-1}$ | $2\times10^{-11}$ | Xu et al.[4] |
| $k_b$ | $m\ s^{-1}$ | $2\times10^{-11}$ | Xu et al.[4] |
| $U$ | V | f($c_p/c_{pmax}$) | Xu et al.[4] |
| $R$ | $J\ mol^{-1}\ K^{-1}$ | 8.314 | - |
| $T$ | K | 293 | - |
| $SoL_{min}$ | 1 | 0.26 | - |
| $SoL_{max}$ | 1 | 0.91 | - |
| $A_s$ | $m^2$ | 0.0024751 | |

**Table S10.** Nomenclature.

| Parameter | Description | Parameter | Description |
|---|---|---|---|
| $\boldsymbol{J}_{\mathrm{p}}$ | Lithium flux | $D_{\mathrm{p}}$ | Active material diffusion coefficient |
| $\boldsymbol{J}_{\mathrm{e}}$ | Lithium ion flux | $D_{\mathrm{e}}$ | Electrolyte diffusion coefficient |
| $\boldsymbol{i}_{\mathrm{p}}$ | Active material current density | η | Overpotential |
| $\boldsymbol{i}_{\mathrm{e}}$ | Electrolyte current density | $i_0$ | Exchange current density |
| $\boldsymbol{i}_{\mathrm{c}}$ | CBD current density | $k_{\mathrm{a}}$, $k_{\mathrm{c}}$ | Reaction rate constants |
| $\phi_p$ | Active material potential | $\alpha_{\mathrm{a}}$, $\alpha_{\mathrm{c}}$ | Transfer coefficients |
| $\phi_e$ | Electrolyte potential | $F$ | Faraday constant |
| $\phi_c$ | CBD potential | $U$ | Open circuit potential |
| $c_{\mathrm{p}}$ | Lithium concentration | $R$ | Universal gas constant |
| $c_{\mathrm{e}}$ | Lithium ion concentration | $f$ | Electrolyte mean molar activity coefficient |
| $c_{\mathrm{p0}}$ | Initial lithium concentration | $t_+$ | Transference number |
| $c_{\mathrm{p,max}}$ | Maximum lithium concentration | $K_{\mathrm{p}}$ | Active material conductivity |
| $c_{\mathrm{e0}}$ | Initial lithium ion concentration | $K_{\mathrm{e}}$ | Electrolyte conductivity |
| $c_{\mathrm{e,ref}}$ | Reference lithium ion concentration | $K_{\mathrm{c}}$ | CBD conductivity |
| $i_0$ | Applied current density | $i_{\mathrm{BV}}$ | Charge transfer rate |
| $V_p$ | Active material volume | $A_{\mathrm{s}}$ | Electrode cross-sectional area |
| $T$ | Temperature | $t_{cc}$ | Current collector thickness |
| $t_{sep}$ | Separator thickness | $L$ | Electrode thickness |
| $\boldsymbol{n}_{\mathrm{i}}$ | Normal vector at given interface in Supplementary table 6 | | |

**References**

[1] Beuse, T., Fingerle, M., Wagner, C., Winter, M. and Börner, M., 2021. Comprehensive Insights into the Porosity of Lithium-Ion Battery Electrodes: A Comparative Study on Positive Electrodes Based on LiNi0. 6Mn0. 2Co0. 2O2 (NMC622). Batteries 7(4), 70.

[2] Valøen, L. O. & Reimers, J. N., 2005. Transport Properties of LiPF6-Based Li-Ion Battery Electrolytes. J. Electrochem. Soc. 152, A882.

[3] Noh, H. J., Youn, S., Yoon, C. S. & Sun, Y. K., 2013. Comparison of the Structural and Electrochemical Properties of Layered Li[NixCoyMnz]O2 (x = 1/3, 0.5, 0.6, 0.7, 0.8 and 0.85) Cathode Material for Lithium-Ion Batteries. J. Power Sources 233, 121–130.

[4] Xu, R. et al., 2019, Heterogeneous Damage in Li-Ion Batteries: Experimental Analysis and Theoretical Modeling. J. Mech. Phys. Solids 129, 160–183.
